# A Comprehensive Analysis of Topiramate and Drug Reaction With Eosinophilia and Systemic Symptoms

**DOI:** 10.7759/cureus.33713

**Published:** 2023-01-12

**Authors:** Amelia Thoms, Patrina Muller, Brigitte Schaufelberger, Eva De La Torre, Steven S Smugar

**Affiliations:** 1 Global Medical Safety, Janssen Research & Development, Raritan, USA; 2 Global Medical Safety, Janssen Research & Development, Horsham, USA; 3 Global Medical Safety, Janssen Research & Development, Titusville, USA

**Keywords:** migraine prophylaxis, seizure disorder, epilepsy, severe cutaneous adverse reaction, drug safety, drug reaction with eosinophilia and systemic symptoms (dress), topiramate

## Abstract

Introduction

Recent publications have described drug reaction with eosinophilia and systemic symptoms (DRESS) with topiramate. Topiramate has been associated with other severe cutaneous adverse reactions, including Stevens-Johnson syndrome, but a relationship to DRESS has not been established. To determine if there is a causal association between topiramate and DRESS, we conducted a comprehensive review of the data in the Janssen Research & Development Global Safety Database (GSD), signaling databases, and the literature.

Methods

The primary data were post-marketing reports of DRESS in the Janssen topiramate GSD (cumulative through 1 July 2022), representing >14,000,000 patient-years (PY) exposure. Cases were reviewed, assigned a Registry of Severe Cutaneous Adverse Reaction (RegiSCAR) score, and assessed for overall contribution of topiramate to DRESS based on temporality, concomitant medications, dechallenge/rechallenge, and baseline patient factors. Statistical disproportionality was evaluated in European Medicines Agency's EudraVigilance (EV) safety database and the United States Food and Drug Administration Adverse Event Reporting System (FAERS). For EV, the overall disproportionality threshold was the lower limit of the 95% confidence interval (CI) for the reporting odds ratio (ROR025) >1 and N ≥5. The overall threshold for FAERS was the Empirical Bayesian Geometric Mean (EBGM) ≥2, lower bound of the 90% CI (EB05) of >1, and N ≥3. To account for the role of concomitant drugs, Empirical Bayes regression-adjusted arithmetic mean (ERAM) scores were calculated, with a threshold ≥2, a lower bound of the 90% CI (ER05) of >1, and N ≥3. An integrated search of major biomedical literature was performed for reports of topiramate and DRESS.

Results

There were 17 reports of DRESS in the GSD (reporting rate 0.12/100,000 PY). RegiSCAR scores ranged from -3 to 7 (average -0.4). No cases met full diagnostic criteria and were highly confounded by the presence of other suspect drugs. Disproportionality scores exceeded thresholds for statistical significance in FAERS (N=72, EBGM=2.06, EB05=1.69), but not in EV (N=33, ROR025=0.79). When accounting for co-administered drugs, ERAM was statistically significant for carbamazepine (4.53), lamotrigine (ERAM=6.54), phenytoin (ERAM=2.91), and zonisamide (ERAM=2.25) exceeding disproportionality thresholds, but the score of topiramate was no longer significant (0.25).

Conclusion

A comprehensive review of all available evidence does not support a causal association between topiramate and DRESS.

## Introduction

Severe cutaneous adverse reactions (SCAR) have been widely reported with anti-seizure medications, including topiramate [1]. Stevens-Johnson syndrome (SJS) and toxic epidermal necrolysis (TEN) are known to be associated with topiramate, as reflected in the product information [2]. Recently, large studies have suggested a possible risk of drug reaction with eosinophilia and systemic symptoms (DRESS) syndrome with topiramate [3,4], but a causal association has not been made previously.

We performed a comprehensive analysis of DRESS with topiramate, including data from the Janssen topiramate global safety database (GSD), the European Medicines Agency EudraVigilance (EV) and Food and Drug Administration Adverse Event Reporting System (FAERS) databases, and a comprehensive review of the literature. To our knowledge, this is the largest such analysis and review.

## Materials and methods

Global Safety Database

The Janssen Research & Development Global Safety Database was searched for all spontaneous and clinical study cases reporting adverse events for topiramate coded to preferred terms (PTs) in the Medical Dictionary for Regulatory Activities (MedDRA; version 25.0) Standardised MedDRA Query (SMQ) drug reaction with eosinophilia and systemic symptoms syndrome (narrow). The search included both medically confirmed and medically unconfirmed cases, and was performed cumulatively through 01 July 2022. The SMQ includes the PTs "drug reaction with eosinophilia and systemic symptoms", "granulomatous T-cell pseudolymphoma", and "pseudolymphoma".

Although Registry of Severe Cutaneous Adverse Reaction (RegiSCAR) scores [5,6] are primarily intended for clinical assessment, we have estimated scores to add context to our analysis. However, since the level of detail provided in post-marketing cases is highly variable and often incomplete, we assigned two levels of imputation for RegiSCAR criteria. For cases providing minimal information beyond the reported adverse reaction (ADR) and little other information, applicable criteria were assigned "0"; none were assigned -1. For reports that provided a reasonable level of case detail, strict criteria assessment was applied; unreported criteria information were assumed to be unknown or negative. E.g., in a minimal-information case, lack of fever information would be scored "0"; for a case that provided a higher level of detail, lack of fever information would be scored "-1". In cases where the information was partially reported vague (e.g., fever reported without a corresponding temperature was annotated as "+/-." Therefore, scores are estimates based on generally limited information and should be interpreted accordingly.

EudraVigilance database

A search of the European Medicines Agency's (EMA) EudraVigilance (EV) safety database was conducted to obtain case counts and reporting odds ratio (ROR) for reports involving topiramate and MedDRA version 25.0, with the PT "drug reaction with eosinophilia and systemic symptoms". EV was accessed directly to obtain a case count and a value for the lower limit of the 95% confidence interval for the ROR (ROR 025). The ROR is the disproportionality statistic used within EV by the EMA. The search used the most currently available cumulative data through 30 June 2022.

The following parameters, based on the EMA recommendations, are used as routine statistical thresholds for identifying drug-event combinations with disproportional reporting within EV: ROR 025 >1, and a cumulative total number of cases ≥5, for topiramate as generic [7].

FAERS database

A Multi-item Gamma Poisson Shrinker (MGPS) data mining run of the Food and Drug Administration Adverse Event Reporting System (FAERS) 2022Q1 database was conducted to obtain case counts and Empirical Bayesian Geometric Mean (EBGM) scores for reports involving topiramate as generic and the MedDRA version 24.1, with the PT "drug reaction with eosinophilia and systemic symptoms". In addition, a Regression-adjusted Gamma Poisson Shrinker (RGPS) analysis was performed in FAERS 2022Q1 for topiramate as generic and the MedDRA, with the PT "drug reaction with eosinophilia and systemic symptoms" to obtain case counts and Empirical-Bayes Regression-adjusted Arithmetic Mean (ERAM) scores. RGPS is a hybrid algorithm that combines the methodology of extended logistic regression and MGPS and adjusts for the effects of polypharmacy.

EBGM and ERAM scores, also referred to as signal scores, serve as a statistical measure of disproportionality of reporting for a given drug-event combination (DEC) and are generated by a computer-assisted algorithm using the empirical Bayes MGPS and RGPS methods. Signal scores represent a comparison between the reporting of a particular event for a specific drug (observed) with that of the same event for all drugs (expected) in the database. The following thresholds were applied for a statistical association between drug and event: for MGPS, an EBGM of ≥2, a lower bound of the 90% confidence interval (EB05) of >1, and a case count of ≥3; for RGPS an ERAM of ≥2, a lower bound of the 90% confidence interval (ER05) of >1, and a case count of ≥3.

In addition, an extension of RGPS was performed that screens for drug-drug interactions for the drug of interest, co-reported suspect drugs, and the event(s) of interest. Calculations of ERAM scores were generated separately for each drug-event pair within a drug-drug-event triplet (i.e., topiramate-co-suspect drug-DRESS). This allowed the identification of co-reported suspect drugs and the respective signal score for the event of interest.

All data mining runs were performed utilizing Empirica™ Signal version 9.1.0.7.374 (Oracle, Austin, Texas).

Literature

An integrated search of the major biomedical literature databases (e.g., BIOSIS Previews (1993 to 2022 week 31), Derwent Drug File (1964 to 2022 week 26), Embase (1974 to 06 July 2022), Medline (1946 to 06 July 2022), and Google Scholar (accessed 07 July 2022)) was performed for all reports/articles relating to the use of topiramate and DRESS cumulative to 07 July 2022.

Only original reports and meta-analyses available in English reporting DRESS with topiramate in humans were considered for inclusion. Non-original data, such as review articles and guidelines, were excluded. The following articles were also excluded: DRESS reported for multiple drugs without specifying topiramate; SCARs reported without distinguishing DRESS separately; cases described in the GSD results; and conference abstracts later published as full manuscripts.

## Results

Global Safety Database

The search of the Janssen Research & Development Global Safety Database retrieved 21 cases coded to the MedDRA SMQ drug reaction with eosinophilia and systemic symptoms syndrome (narrow). Of these 21 cases, one was a duplicate, and three contained multiple unidentifiable patients, and are not discussed further. The remaining 17 unique cases are described in Table 1, along with estimated RegiSCAR scores. No cases were reported from company-sponsored clinical studies.

**Table 1:** Case details Key: Atyp – atypical lymphocytes; DRESS - Drug Reaction With Eosinophilia and Systemic Symptoms; Dur – duration; Eos – eosinophilia; Lym – lymphadenopathy; Excl – exclusion; Org – organ involvement; RegiSCAR - European Registry of Severe Cutaneous Adverse Reaction ᵃLatency has been calculated from the drug initiation to the onset of event of interest. ᵇScores are approximate. Please refer to methods

Case	Age (Y) /Sex	Relevant concomitant drugs (latency [d])ᵃ	Fever	Lym	Eos	Atyp	DRESS rash	DRESS biopsy	Org	Dur ≥15 days	Excl other causes	RegiSCAR Scoreᵇ	Dechallenge
1	12/F	Topiramate (26) Clobazam (26) Valproate (26)	-1	0	+/-	0	-1	0	1	-1	0	-2	Y
2 [8]	35/M	Topiramate (~70)	-1	0	0	0	-1	1	0	-1	0	-2	Y
3	43/F	Topiramate (10-17) Gabapentin (NA) Baclofen (NA) Zolpidem (NA)	-1	0	0	0	-1	0	0	-1	0	-3	Y (topiramate and gabapentin)
4	74/M	Topiramate (13) Lacosamide (NA) Levetiracetam (NA)	-1	0	2	0	-1	0	0	-1	0	-1	NR
5	1.6/M	Topiramate (6) Fosfomycin (<1, 6) Meropenem (6)	-1	0	0	0	-1	0	1	-1	0	-2	NR
6	NR/NR	Topiramate (33) Zonisamide (33) Lamotrigine (>33) Carbamazepine (>33)	-1	0	0	0	-1	0	0	-1	0	-3	Y (Topiramate and zonisamide)
7 [9]	25/M	Topiramate (14) Phenytoin (>14)	+/-	1	2	1	1	1	1	0	0	7	Y
8	59/F	Topiramate (42) Lacosamide (42)	-1	2	0	0	1	0	1	-1	0	3	Y
9	14/M	Carbamazepine (14-21) Amoxicillin (14-21) Topiramate (NA – started after reaction)	-1	0	0	0	-1	-1	1	-1	0	-3	NA
10	88/F	Topiramate (28-49) Carbamazepine (28-49)	-1	0	1	0	1	0	1	-1	0	1	Y (topiramate and carbamazepine)
11 [10]	15/M	Topiramate (>90) Valproate (>90) Phenytoin (NA [<90])	+/-	0	0	0	1	0	1	-1	0	1	Y (Phenytoin)
12 [11]	25/F	Topiramate (NR)	0	0	0	0	0	0	0	0	0	0	NR
13	44/M	Topiramate (NR) Phenytoin (NR)	0	0	0	0	0	0	0	0	0	0	NR
14	NR/NR	Topiramate (NR) Phenytoin (NR)	0	0	0	0	0	0	0	0	0	0	NR
15	13/M	Topiramate (3) Phenobarbital (8) Midazolam (NR) Phenytoin (NR) Thiopental (NR)	+/-	0	0	+/-	0	0	1	-1	0	0	NA
16	60/F	Topiramate (NR)	0	0	0	0	0	0	0	0	0	0	NR
17 [12]	55/F	Topiramate (NR) Tramadol (NR) Hydroxyzine (NR) Meloxicam (NR)	-1	0	0	0	-1	1	0	-1	0	-2	NR

 

EudraVigilance database

Table 2 lists the case count and the regional and global ROR 025 for the MedDRA PT "drug reaction with eosinophilia and systemic symptoms" reported with topiramate compared against all drugs in EV.

**Table 2:** Case counts and ROR 025 in EudraVigilance for drug reaction with eosinophilia and systemic symptoms with topiramate through 30 June 2022 Bolded text - score is disproportionately reported DEC - drug-event combination; MedDRA - Medical Dictionary for Regulatory Activities; N - total number of cases; ROR - reporting odds ratio ROR 025 All - lower bound of 95% confidence interval of the ROR for the concerned DEC, using all other DECs available in the database as reference ROR 025 by geographical regions - the columns named ROR(-) Europe, North America, Japan, Asia, rest of the world contain the 95% confidence interval lower bound of the ROR for the spontaneous (excluding litigation) cases related to the concerned DEC originated respectively in those regions using all other DECs originated in those regions as background distribution

MedDRA preferred term	ROR 025 Europe (N)	ROR 025N America (N)	ROR 025 Japan (N)	ROR 025 Asia (N)	ROR 025 rest of world	ROR 025 All (N)
Drug reaction with eosinophilia and systemic symptoms	0.27 (7)	1.03 (10)	0.47 (3)	1.46 (12)	0.00	0.79 (33)

The ROR 025 in two of the five regions (North America and Asia) meet the threshold for disproportionality. The ROR 025 in the remaining three regions and the global value (ROR 025 All) do not meet the threshold for disproportionality.

FAERS database

The case count, EBGM score, and ERAM score for the MedDRA PT "drug reaction with eosinophilia and systemic symptoms" reported with topiramate compared against all drugs in the FAERS database are presented in Table 3.

**Table 3:** Case counts and EBGM and ERAM scores in FAERS through 2022Q1 for drug reaction with eosinophilia and systemic symptoms with topiramate Bolded text - score is disproportionately reported EBGM - Empirical Bayesian Geometric Mean; ERAM - Empirical-Bayes Regression-adjusted Arithmetic Mean; FAERS - Food and Drug Administration Adverse Event Reporting System; MedDRA - Medical Dictionary for Regulatory Activities; MGPS - Multi-Item Gamma Poisson Shrinker; N - Number of cases; Q - Quarter; RGPS - Regression-adjusted Gamma Poisson Shrinker EB05 - Lower limit of 90% confidence interval for EBGM; ER05 - Lower limit of 90% confidence interval for ERAM; there is approximately a 5% probability that the ERAM lies below this value

MedDRA preferred term	FAERS 2022Q1				
	N	MGPS		RGPS	
		EBGM	EB05	ERAM	ER05
Drug reaction with eosinophilia and systemic symptoms	72	2.06	1.69	0.25	0.205

The signal scores (EBGM, EB05) for the MGPS are elevated and meet the threshold for disproportionality. However, when accounting for polypharmacy the signal scores (ERAM, ER05) are below the threshold for disproportionality.

An extension of RGPS was performed to screen for drug-drug-event triplets comprising topiramate, a co-reported suspect drug, and the MedDRA PT "drug reaction with eosinophilia and systemic symptoms". Table 4 shows the following for each drug-drug-event triplet: ERAM values for topiramate and the co-suspect drug, the total number of cases co-reporting topiramate and the co-suspect drug (drug one + drug two), and the number of cases reporting the drug-drug-event triplet including topiramate, the co-suspect drug, and DRESS (drug one + drug two + event).

No ERAM scores met the threshold for disproportionality for topiramate, but the threshold for disproportionality was met for the following co-suspect drugs: carbamazepine, lamotrigine, phenytoin, and zonisamide.

**Table 4:** Case counts and ERAM scores in FAERS through 2022q1 for drug reaction with eosinophilia and systemic symptoms with topiramate and co-suspect drugs using RGPS drug-drug interaction computation Bolded text - score is disproportionately reported. DRESS - drug reaction with eosinophilia and systemic symptoms; FAERS - Food and Drug Administration Adverse Event Reporting System; MedDRA - Medical Dictionary for Regulatory Activities; Q - Quarter; RGPS - Regression-adjusted Gamma Poisson Shrinker; N for drug 1 + drug 2 = number of reports for drug 1 and drug 2 as co-reported suspect drug in FAERS; N for drug 1 + drug 2 + event = number of cases reporting drug 1, drug 2 (co-reported suspect drug), and event in FAERS

Drug 1	Drug 2	FAERS 2022Q1			
		ERAM for drug 1	ERAM for drug 2	N for drug 1 + drug 2	N for drug 1 + drug 2 + event
Topiramate	Carbamazepine	0.25	4.53	1372	5
Topiramate	Clobazam	0.25	0.27	961	2
Topiramate	Lamotrigine	0.25	6.54	1883	13
Topiramate	Levetiracetam	0.25	1.00	2302	11
Topiramate	Phenobarbital	0.25	1.41	797	7
Topiramate	Phenytoin	0.25	2.91	948	28
Topiramate	Valproic acid	0.25	0.99	2561	10
Topiramate	Zonisamide	0.25	2.25	536	2

Literature review

A total of 132 publications were retrieved. Of these, 126 were excluded from detailed analysis for the following reasons: review articles (N=68); DRESS not reported with topiramate (N=43); DRESS reported for multiple drugs without specifying topiramate, or reported SCARs in general without reporting DRESS separately (N=7); cases described in the Janssen Research & Development Global Safety Database results (N=3); full text in English not available (N=2, both single case reports); conference abstracts later published as full manuscripts (N=2); animal case report (N=1)

The remaining six publications describing DRESS with topiramate are described below.

Chung et al. [3] performed an analysis of SCAR with AEDs in a nationwide claims database in Korea (N=302,014). To focus on SCAR causeed by monotherapy, the authors stopped analyzing data for a patient when the AED was switched or another antiepileptic drug (AED) was used concomitantly. A total of 242 patients had SCARs, of whom 88 received topiramate. Of these 88 cases, 71 reported DRESS, with a calculated incidence rate (IR) of 320 per 1,000,000 patient-years (PY). Notably, 18 (20.4%) patients received concomitant antibiotics or nonsteroidal anti-inflammatory drugs, which were associated with increased IRs in a separate analysis. Overall, the IR for topiramate was lower than that observed for carbamazepine, phenytoin, lamotrigine, zonisamide, and levetiracetam (640, 4700, 2630, 1180, and 650 per 1,000,000 PY, respectively). The primary and significant limitation of this study is that diagnoses were based on ICD-10 codes and not formally adjudicated. Therefore, it is unknown whether DRESS and other SCARs met established diagnostic criteria.

Kim et al. [4] performed an analysis of SCAR with AEDs using the Korea Institute of Drug Safety and Risk Management - Korea Adverse Event Reporting System. Of 2,942 reports, 64 (2.2%) involved topiramate, 1 (1.6%) of which reported DRESS. Six drugs had ≥5 cases of DRESS: carbamazepine (60/512; 11.7%), lamotrigine (12/699; 1.7%, valproic acid (11/677; 1.6%), oxcarbazepine (8/320; 2.5%), levetiracetam (6/181; 3.3%), and phenytoin (5/158; 3.2%). As with the previous study, the major limitation was that SCAR and DRESS were not formally diagnosed. In addition, it is not clear whether the authors accounted for concomitant AED use.

A conference abstract by Renda et al. [13] described a disproportionality assessment of DRESS using EudraVigilance. The ROR for topiramate (1.65; 95% CI 1.20-2.27) was disproportionately reported. It is notable that the ROR for topiramate was the second-lowest statistically significant result among the 17 drugs with significant results. By comparison, the ROR for zonisamide (ROR: 15.70; 95% CI 12.94-s19.06), phenytoin (ROR: 14.77; 95% CI 13.45-16.23), phenobarbital (ROR: 13.89; 95% CI 12.18-15.85), carbamazepine (ROR: 13.81; 95% CI 13.10-14.55) and lamotrigine (ROR: 13.57; 95% CI 12.59-14.63) were substantially higher. As with the above studies, DRESS was not formally adjudicated. In addition, as this was a conference abstract with limited information, including the full study methodology, a definitive assessment cannot be made.

Pereira De Silva et al. [14] described DRESS (RegiSCAR ≥5) in eight patients treated with AEDs. Among these, one patient experienced DRESS 45 days after starting lamotrigine. The patient had also been receiving topiramate and valproic acid, but these were not considered suspect drugs due to long-term use (540 days each).

Santiago et al. [15] evaluated 56 patients with DRESS (RegiSCAR ranged from two to eight). A total of 33 patients had AED-associated DRESS, of whom one was receiving topiramate. This patient also had a positive patch test, which the authors note was only the second positive patch test for topiramate reported in the literature. No further information is available about this case. The authors stated RegiSCAR scores ranged from two to eight. Therefore, although the RegiSCAR score is not known for this particular patient, it can be assumed it is at least a two, and therefore a DRESS diagnosis is considered at least possible.

Dahl et al. [16] described a two-year-old girl with febrile-illness-related epilepsy syndrome, initially treated with broad-coverage antibiotics (ampicillin, cefotaxime, ceftriaxone, and vancomycin), lorazepam, fosphenytoin, propofol, midazolam, fentanyl, phenobarbital, lacosamide, valproate, topiramate, felbamate, clonazepam, and levetiracetam. Specific details were not provided regarding the initiation and duration of topiramate or other AEDs. Approximately three weeks later, the patient developed a reticular rash that evolved into a morbilliform rash generalized to her trunk, face, and extremities. Laboratory studies showed eosinophilia, increased aspartate transferase (AST), alanine transaminase (ALT), and both direct and total bilirubin (values not provided). Cytomegalovirus, Epstein-Barr virus, and human herpesvirus-6 were negative. Skin biopsy showed lichenoid interface dermatitis. This patient received multiple AEDs and antibiotics, the timing of which is unclear, but it does not seem the patient was receiving topiramate at the time DRESS started. The use of multiple drugs with a propensity for DRESS and simultaneous dechallenges preclude clear causal attribution. Nevertheless, the closer temporal association of the other drugs to DRESS and their known association with DRESS suggest they are more likely culprits. The RegiSCAR score for this case is 0.

## Discussion

None of the cases from the GSD represented a sentinel case. Most cases lacked specific clinical and laboratory evidence of DRESS and were highly confounded by concomitant medications with a known risk of DRESS. RegiSCAR scores [5,6] were exceedingly low, ranging from -3 to 7 (mean -0.4). Since RegiSCAR scoring is used for clinical diagnosis, low scores based on post-marketing reports are perhaps not surprising. Nevertheless, two of the five case reports from the literature had scores of -2. This further highlights the caution that must be taken in assigning causality to diseases with complex diagnostic criteria. Although there were cases with RegiSCAR scores ≥2, suggesting a diagnosis of DRESS is at least possible, these cases were nonetheless highly confounded by factors such as unlikely latency and concomitant medications to identify topiramate as the culprit drug.

The results from EV did not meet the overall threshold (ROR 025 ALL= 0.79) for disproportionality. However, there were some regions in Asia (ROR 025= 1.46) and North America (ROR 025= 1.03) where the ROR 025 reached disproportionality. Regional differences in signal scores could be due to regional differences in reporting, distribution, prescribing of co-reported drugs, or in pharmacogenetic susceptibility (e.g., polymorphism). Indeed, it is notable that a plurality of cases (N=7) from the Company Global Safety Database were from Asia, and pharmacogenomic studies have shown human leukocyte antigen allele associations with SCAR in Asian populations [17,18].

The results from FAERS showed a statistical association between topiramate and DRESS. However, after using RGPS, a logistic regression analysis, the statistical association between topiramate and DRESS was no longer significant, whereas concomitant carbamazepine, lamotrigine, phenytoin, and zonisamide were shown to have statistical significance, consistent with previous findings [1,19]. This data suggests that topiramate may be an innocent bystander.

The literature was weakly supportive of a causal association between topiramate and DRESS. The three large studies had no formal DRESS diagnosis [3,4,13], and the remaining three publications each reported one case of DRESS with topiramate, one of which occurred after lamotrigine was introduced to long-term topiramate and valproate therapy and one in the setting of multiple AEDs and antibiotics [14-16].

Overall, considering 17 spontaneous cases and more than 14,000,000 PY exposure since product approval in 1995, the rate of spontaneously reported DRESS with topiramate is exceedingly rare at 0.12/100,000 PY and substantially lower than the background rate of 0.9 to 2/100,000 [20]. In addition, no cases were reported in Janssen Research & Development-sponsored clinical trials (N>23,000).

The primary limitation of this analysis is the nature of post-marketing reports, which are often incomplete despite follow-up attempts with reporters. This also highlights the caution in assigning causality to diseases with complex diagnostic criteria from signaling databases such as EV and FAERS. The use of large signaling databases is also limited by a number of factors, including coding practices and duplicate cases. Both are limited insofar by the lack of a true denominator. For reports in the GSD, the reporting rate is estimated on sales and distribution data and may not reflect actual usage. Finally, adverse reaction under-reporting is well-known and estimated to be as high as 98% [21]; therefore, the true event rate is likely higher.

## Conclusions

Overall, in considering the nature of the cases and extent of exposure in both the post-marketing and clinical settings, as well as the results from two large health authority safety databases and the literature, DRESS is unlikely to be causally associated with topiramate use.
